# Effects of dietary nano-selenium and lysophospholipid supplementation on sperm quality and testicular histomorphometry in Moghani ram

**DOI:** 10.1016/j.vas.2026.100733

**Published:** 2026-06-11

**Authors:** Leila Aghazadeh, Farzad Mirzaei Aghjeh-gheshlagh, Hossein Jahani-Azizabadi, Bahman Navid-Shad, Ali Kalantari-Hesari, Hamid Staji

**Affiliations:** a Department of Animal Sciences, Faculty of Agriculture & Natural Resources, University of Mohaghegh Ardabili, Ardabil, Iran; b Department of Animal Sciences, Faculty of Agriculture, University of Kurdistan, Sanandaj, Kurdistan, Iran; c Department of Animal Sciences, Faculty of Agriculture, University of Guilan, Rasht, Iran; d Department of Pathobiology, Faculty of veterinary science, Bu Ali Sina University, Hamedan, Iran; e Department of Pathobiology, Faculty of Veterinary Medicine, University of Semnan, Semnan, Iran

**Keywords:** Histomorphometric, Lysophospholipid, Nano-selenium, Semen, Ram

## Abstract

This study aimed to evaluate the effects of dietary nano-selenium (nano-Se) and lysophospholipid (LPL) supplementation on sperm quality, fertility-related indices, and histomorphometric characteristics of testicular tissue in Moghani rams. Thirty-six rams (42.2 ± 0.07 kg) were randomly assigned to six experimental treatments for 90 days (15 days adaptation and 75 days experimental period). Treatments included: (1) control (basal diet supplemented with 0.5 mg/kg DM organic selenium), (2) 0.05% LPL, (3) 0.1% LPL, (4) 0.5 mg nano-Se/kg DM, (5) 0.05% LPL + nano-Se, and (6) 0.1% LPL + nano-Se.The results showed that treatment 6 significantly improved testicular histomorphometric parameters, including increased epithelial height, Sertoli and Leydig cell numbers, and enhanced tubular differentiation, spermiogenesis, and repopulation indices (P < 0.05). Additionally, the proportion of degenerated seminiferous tubules and lumen diameter was significantly reduced in this group (P < 0.05).In contrast, treatment 5 resulted in superior sperm functional characteristics, including higher total sperm count, improved viability, and increased DNA integrity (P < 0.05). Treatment 6 also reduced the percentage of histone-immature spermatozoa (P < 0.05), indicating improved sperm maturation. No significant differences were observed among treatments in semen volume, seminiferous tubule diameter, or cauda epididymal epithelial height (P ≥ 0.05).In conclusion, higher levels of LPL combined with nano-Se primarily enhanced testicular structure and spermatogenic activity, whereas lower levels of LPL with nano-Se were more effective in improving sperm functional quality. These findings suggest a dose-dependent role of lysophospholipids in modulating reproductive performance in rams.

## Highlights

• The use of 0.1% LPL + 0.5 mg of nano-Se improved spermatogenesis and spermiogenesis processes in rams.
• The inclusion of nano-Se and LPL significantly increased epithelial height, the number of Sertoli cells and the number of Leydig cells.
• The values of the tubular differentiation index, repopulation index, and spermiogenesis index increased by nano-Se and LPL.
• Diets containing nano-Se and LPL significantly enhanced total sperm count, sperm viability, and the percentage of sperm with intact DNA.

## 1 Introduction

Reproductive efficiency is a critical determinant of productivity in sheep production systems, with male fertility playing a central role. Among the major factors impairing male reproductive performance, oxidative stress—resulting from excessive production of reactive oxygen species (ROS)—can damage sperm lipids, proteins, and DNA, leading to reduced motility, viability, and fertilization capacity (Ansari et al., 2021; Evans et al., 1996; Tvrdá et al., 2011). Therefore, nutritional strategies aimed at enhancing antioxidant mechanisms are increasingly recognized as effective approaches to improve semen quality and reproductive outcomes. Recent studies further highlight the importance of controlling oxidative stress to preserve sperm DNA integrity and overall fertility in livestock (Agarwal et al., 2021; Dutta et al., 2019).

Selenium (Se) is an essential trace element with a well-established role in male reproduction, primarily through its incorporation into selenoproteins such as glutathione peroxidase, which protect spermatozoa from oxidative damage (Hosnedlova et al., 2018; Wu et al., 1973). Advances in nanotechnology have introduced nano-selenium (nano-Se), which exhibits higher bioavailability, improved tissue retention, and lower toxicity compared to conventional selenium sources (Khalil et al., 2019; Patra and Lalhriatpuii, 2020). Emerging evidence suggests that nano-Se can enhance testicular function, antioxidant status, and sperm quality in livestock species (Arshad et al., 2021; Han et al., 2021; Yuan et al., 2024). However, its potential interactions with dietary compounds that influence nutrient absorption and membrane functionality remain insufficiently understood.

Lysophospholipids (LPLs) are bioactive lipid molecules known to improve nutrient digestibility, membrane permeability, and lipid metabolism (Joshi et al., 2006). Beyond their nutritional role, LPLs have been associated with improved reproductive performance, including enhanced spermatogenesis, sperm membrane integrity, and fertilization processes (Boontiam et al., 2017; Ishii et al., 2001). Their amphiphilic structure enables modulation of cell membrane dynamics, which is particularly relevant for sperm maturation and function. Recent studies also suggest that LPL supplementation may enhance nutrient utilization efficiency and cellular signaling pathways linked to reproductive performance (Liu et al., 2023; Zhao et al., 2017). Despite these promising effects, limited information is available regarding the combined influence of LPLs and nano-Se on male reproductive traits.

Therefore, the present study was designed to evaluate the effects of dietary nano-Se and lysophospholipid supplementation, alone or in combination, on sperm characteristics, fertility-related indices, and histomorphometric parameters of testicular tissue in Moghani rams. It was hypothesized that the combined use of nano-Se and LPLs would exert complementary or synergistic effects by enhancing antioxidant capacity and improving both structural and functional aspects of spermatogenesis.

## 2 Materials and methods

### 2.1 Animals, experimental design, diet, and feed analysis

The present study was conducted over a 90-day period (comprising a 15-day adaptation phase and a 75-day sampling phase) from July to September 2022 at the research farm of Mohaghegh Ardabili University, located in Ardabil Province, Iran. A total of thirty-six of the same age Moghani rams with an average body weight of 42.2 ± 0.07 kg were used in this experiment. The animals were randomly assigned to six experimental treatments (n = 6 per treatment). The experimental groups were as follows: 1: Control diet: Basal diet supplemented with 0.5 mg/kg DM organic selenium, representing the standard farm practice, 2: Control diet + 0.05% LPLs, 3: Control diet + 0.1% LPLs, 4: Basal diet + 0.5 mg nano-Se per kg of DM, 5: Basal diet + 0.05% LPL + 0.5 mg nano-Se per kg of DM, 6: Basal diet + 0.1% LPL + 0.5 mg nano-Se per kg of DM.

The nano-Se and organic selenium used in this study were manufactured by Pishgaman Nano Materials Company, Mashhad, Iran. The lysophospholipid complex used in this study was obtained from soybean lecithin by the exclusive proprietary technology developed by the UK-based company Pathway-Intermediates (Lipidol®; EASY BIO System Inc., Seoul, South Korea).. This product contains 6% lysophospholipids, 43% other lipids (phospholipids and triglycerides), and 50% carrier, which serve as active components and absorption enhancers.

The experimental diets were formulated using CNCPS software (version 1.0.21) to meet the nutritional requirements of growing male lambs with the specified initial weight. The rams were housed individually and received restricted feeding twice daily at 9:00 and 17:00. The diets were offered as a total mixed ration (TMR), and animals had free access to clean drinking water throughout the experimental period (Table 1).

**Table 1:** Ingredients of the experimental diets (% of DM).

Ingredients (% of DM)	Treatments *					
	1	2	3	4	5	6
Alfalfa hay	29.5	29.45	29.4	29.5	29.45	29.4
Barley grain	31.5	31.5	31.5	31.5	31.5	31.5
Wheat bran	10	10	10	10	10	10
Corn meal	22.5	22.5	22.5	22.5	22.5	22.5
Soybean meal	3	3	3	3	3	3
Bicarbonate sodium	0.5	0.5	0.5	0.5	0.5	0.5
Calcium phosphate	0.5	0.5	0.5	0.5	0.5	0.5
Dicalcium phosphate	0.5	0.5	0.5	0.5	0.5	0.5
Salt	0.5	0.5	0.5	0.5	0.5	0.5
Vitamin supplements and Minerals	1	1	1	1	1	1
Organic Selenium	0.5	0.5	0.5	-	-	-
Nano-Se	-	-	-	0.5	0.5	0.5
Lysophospholipids	-	0.05	0.1	-	0.05	0.1

⁎ Treatments: 1. Control (basal diet + 0.5 mg of organic selenium per kg of DM), 2. Control diet + 0.05% Lysophospholipid, 3. Control diet + 0.1% Lysophospholipid, 4. Basal diet + 0.5 mg Nano selenium per kg of DM, 5. Basal diet + 0.05% Lysophospholipid + 0.5 mg of Nano selenium per kg of DM, 6. Basal diet + 0.1% Lysophospholipid to + 0.5 mg of Nano selenium per kg of DM.

### 2.2 Sampling procedures and measurements

#### 2.2.1 Body weight and scrotal circumference

On the final day of the experiment, one day before slaughter, final body weight (kg) and scrotal circumference (cm) were measured using a digital scale and a tape measure, respectively.

#### 2.2.2 Left testis weight

At the end of the experimental period, the rams were slaughtered, and the testis was collected for histological analysis. The left testis weight (kg) was measured using a precision scale (Jit and Sanjeev, 1991).

#### 2.2.3 Semen collection

In the sixth week of the experimental period, the rams (of the same age) were trained for semen collection using an artificial vagina. For adaptation purposes, each ram was ejaculated three to four times per week (Camps et al., 1981). Semen samples were collected from each rams during the final three weeks of the study and each ram was ejaculated one time per week. Immediately after collection, semen volume, sperm count and concentration, viability (using eosin-nigrosin staining), and motility were evaluated.

#### 2.2.4 Sperm count

For sperm counting, a 1:20 dilution of semen was prepared by mixing 10 µL of semen with 190 µL of distilled water. Then, 10 µL of the diluted sample was placed on a Neubauer hemocytometer, and sperm cells were counted in the 25 central squares under a microscope. The final count was multiplied by 5 × 10⁶ to estimate sperm concentration (Rezvanfar et al., 2008; Rezvanfar et al., 2013).

#### 2.2.5 Sperm motility

Sperm samples were collected from the epididymis and placed in HTF culture medium (placed in an incubator at 37.2°C, 5% Co_2_, and 70% humidity). After the sperm were released from the epididymal caudal tissue, a volume of 20 microliters of the medium containing sperm was transferred onto a Neubauer chamber on a hot plate (37.02°C). Then, at least 400 sperm were counted in 20 to 30 microscopic fields for each sample. A cell counter was used to count the number of motile and immobile sperm. The method of counting motile sperm was such that motile sperm that left the field were counted, but sperm that entered the field were not counted. Also, motile sperm that had an active and forward movement were considered motile, and the rest of the sperm were counted as non-motile sperm. Finally, the ratio of motile to non-motile sperm was reported. Sperm motility was assessed by counting the number of motile and non-motile spermatozoa in 5 to 6 microscopic fields (Rezvanfar et al., 2008; Rezvanfar et al., 2013).

#### 2.2.6 Semen volume

Semen volume was measured using a graduated conical tube with 0.1 mL accuracy (Rezvanfar et al., 2008; Rezvanfar et al., 2013).

#### 2.2.7 Sperm viability

Sperm viability was evaluated using eosin-nigrosin staining. A 20 µL semen sample was mixed with 20 µL eosin solution, and after 20–30 seconds, 20 µL of nigrosin stain was added. A smear was prepared from the mixture and air-dried. The percentage of live (unstained) and dead (red-stained) sperm was determined under a light microscope at 400 × magnification (Rezvanfar et al., 2008; Rezvanfar et al., 2013).

#### 2.2.8 Sperm histone maturity

Histone protein is a protein that is degraded during sperm maturation and can be detected if the sperm is not fully mature. By staining the remaining histones using aniline blue staining, mature and immature sperm can be distinguished from each other. Sperm samples were centrifuged and then slide spread was prepared. Then, sperm samples were fixed using acetone. Finally, the slides were stained with aniline blue and the number of sperm with histone maturity was counted in 400 sperm.

Aniline blue staining was used to assess sperm histone maturity. Spermatozoa with immature histones stained dark blue due to their affinity to aniline blue. After smearing and air-drying, the slides were fixed with acetone and stained with aniline blue for 7 min. The slides were then examined under a light microscope, and the percentages of mature and immature sperm based on histone content were calculated (Rezvanfar et al., 2008; Rezvanfar et al., 2013).

#### 2.2.9 Sperm DNA damage

If the DNA strands are not properly closed, these strands can be traced as single-stranded and unstrained. Using acridine orange staining, sperm with single-stranded DNA can be traced. Sperm samples were centrifuged and then slide spreads were prepared. Then, the sperm samples were fixed using acetone. Finally, the slides were stained with acridine orange and 400 sperm were counted.

Acridine orange staining was employed to assess DNA damage in sperm cells. This fluorescent dye differentiates intact double-stranded DNA (green fluorescence) from damaged single-stranded DNA (yellow to red fluorescence) under a fluorescence microscope. After smear preparation and fixation with Carnoy's solution, samples were stained for 10 minutes and then examined using a 460 nm filter (Rezvanfar et al., 2008; Rezvanfar et al., 2013).

#### 2.2.10 Testicular histomorphometry

At the end of the experimental period, the rams were slaughtered, and the testis was collected for histomorphometry analysis. The samples were placed in 10% buffered formalin solution for fixation. For light microscopy examination, testicular tissue samples were fixed in 10% buffered formalin. After routine processing and paraffin embedding, 5 μm sections were prepared using a rotary microtome. Histological and morphometric analyses were conducted on the left testis, evaluating the capsule, Leydig tissue, and seminiferous tubules for structural integrity and abnormalities, following the method of Suvarna et al. (2012). Hematoxylin and eosin staining was used for morphometric assessment of parameters including: Seminiferous tubule diameter, Tubular epithelial height, Lumen diameter, Number of Sertoli and Leydig cells, Tubular Differentiation Index (TDI): percentage of tubules containing ≥4 layers of differentiated cells from type A spermatogonia, Spermiogenesis Index (SI): percentage of tubules containing sperm and Repopulation Index (RI): ratio of active (type B) to inactive (type A) spermatogonia. Measurements were conducted using a Dino-Lite digital microscope camera and Dino-Capture software (version 2). From every five sections per testis, one section was selected, and parameters were assessed at 35 μm intervals of tissue (Hesari et al., 2015).

In the present study, due to limited funding, measurements of reproductive hormones (e.g., testosterone), oxidative stress markers, antioxidant biomarkers, and advanced semen quality indices were not conducted."

### 2.3 Statistical analysis

Data were analyzed using a completely randomized design with a 2 × 3 factorial arrangement (two levels of selenium source and three levels of lysophospholipid). Statistical analysis was performed using the GLM procedure of SAS (version 9.4; SAS Institute, Cary, USA). The model included the main effects of lysophospholipid, selenium, and their interaction. When significant effects were detected (P < 0.05), means were compared using Tukey’s multiple range test. Data normality was assessed using the Kolmogorov–Smirnov test. Results are presented as mean ± standard error.

The experimental model used in this study was:

$$y_{ijk}=\mu +A_{i}+B_{j}+C_{k}+{\left(AB\right)}_{ij}+{\left(AC\right)}_{ik}+{\left(BC\right)}_{jk}+{\left(ABC\right)}_{ijk}+e_{ijkl}$$

Where: y_ijk_ = the (l)-th observation at level (i) of factor A, level (j) of factor B, and level (k) of factor C.

µ = overall mean of the population.

A_i_ = effect of the (i)-th level of factor A.

B_j_ = effect of the (j)-th level of factor B.

C_k_ = effect of the (k)-th level of factor C

(AB)_ij_ = interaction effect between the (i)-th level of factor A and the (j)-th level of factor B.

(AC)_ik_ = interaction effect between the (i)-th level of factor A and the (k)-th level of factor C.

(BC) _jk_ = interaction effect between the (j)-th level of factor B and the (k)-th level of factor C.

(ABC)_ijk_ = three-way interaction effect among the (i)-th level of factor A, (j)-th level of factor B, and (k)-th level of factor C. e_ijkl_ = experimental error.

This model accounts for main effects, two-way interactions, and the three-way interaction among the factors.

#### 2.3.1 2.X. power analysis

A post hoc power analysis was conducted to evaluate the adequacy of the experimental design based on one of the key reproductive traits (sperm concentration). The analysis was performed using G*Power software (version X.X), considering the observed effect size, sample size (n = 6 per treatment), and a significance level of P < 0.05. The calculated statistical power (1 – β) was used to assess the probability of detecting biologically meaningful differences among treatments.

## 3 Results

### 3.1 Body weight, scrotal circumference, and testis weight

There was a significant increase in body weight in Treatment 6 compared to the other experimental treatments (P < 0.05) (Table 2). However, the highest body weight (53.7 kg) was observed in Treatment 4, while the lowest body weight (51.1 kg) was recorded in Treatment 1 (control). Testis weight showed a significant increase in Treatment 5 compared to the other experimental treatments (P < 0.05) (Table 2). The highest testis weight (259.2 g) was observed in Treatment 5, whereas the lowest value (195.8 g) was recorded in Treatment 2. Similarly, scrotal circumference was significantly higher in Treatment 1 (control) compared to the other experimental treatments (P < 0.05) (Table 2). The maximum scrotal circumference (29.5 cm) was recorded in Treatment 1 (control), while the minimum value (27.5 cm) was observed in Treatment 2.

**Table 2:** Effects of selenium sources and Lysophospholipid on average final body weight, left testis and scrotal circumference of rams.

	Treatments *					P-value				
	1	2	3	4	5	6	SEM	Lyso	Nano-Se	Lyso* Nano-Se
Final Body weight (kg)	51.1^b^	51.3 ab	52.9 ab	53.7^a^	53.5 ab	53.2 ab	0.39	0.69	0.03	0.43
Scrotal circumference (cm)	29.5^a^	27.5^b^	27.6^ab^	27.8 ab	28.6 ab	27.8 ab	0.23	0.24	0.80	0.04
Left testes weight (g)	233.3 ab	195.8^b^	247.5 ab	209.2 ab	259.2^a^	223.3 ab	6.91	0.66	0.69	0.01

⁎ Treatments: 1. Control (basal diet + 0.5 mg of organic selenium per kg of DM), 2. Control diet + 0.05% Lysophospholipid, 3. Control diet + 0.1% Lysophospholipid, 4. Basal diet + 0.5 mg Nano selenium per kg of DM, 5. Basal diet + 0.05% Lysophospholipid + 0.5 mg of Nano selenium per kg of DM, 6. Basal diet + 0.1% Lysophospholipid to + 0.5 mg of Nano selenium per kg of DM.

a,b Means within a row with different superscripts are significantly different (P<0.05).

### 3.2 Histological observations

#### 3.2.1 Testicular histology

**Fig. 1:**
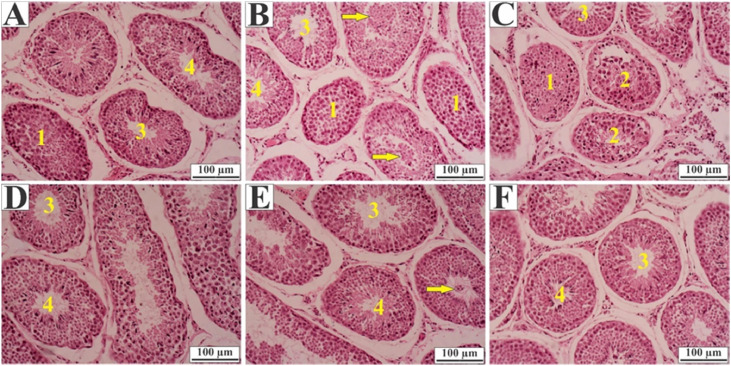
Effects of selenium sources and Lysophospholipid on histological sections of testis tissue, stained with hematoxylin and eosin, × 100 magnification. A: Treatment 1. Control (basal diet + 0.5 mg of organic selenium per kg of DM); B: Treatment 2. Control diet + 0.05% Lysophospholipid; C: Treatment 3. Control diet + 0.1% Lysophospholipid; D: Treatment 4. Basal diet + 0.5 mg Nano selenium per kg of DM; E: Treatment 5. Basal diet + 0.05% Lysophospholipid + 0.5 mg of Nano selenium per kg of DM; F: Treatment 6. Basal diet + 0.1% Lysophospholipid to + 0.5 mg of Nano selenium per kg of DM. 1: Immature seminiferous tubule; 2: Degenerated seminiferous tubule; 3: TDI-positive tubule; 4: SI-positive tubule; arrow: sloughed immature spermatogenic cells in the central lumen.

Histological examination of testicular sections in Treatment 1 revealed that a limited number of seminiferous tubules appeared immature and solid (Fig. 1A). In Treatment 2, a greater number of immature seminiferous tubules were observed, and in some tubules, sloughing of immature spermatogenic cells into the central lumen was evident (Fig. 1B). Treatment 3 exhibited an even higher number of immature seminiferous tubules, accompanied by pronounced sloughing of immature spermatogenic cells into the lumen in several tubules. Additionally, some degenerated tubules were observed (Fig. 1C). In Treatments 4 and 5, only a few immature seminiferous tubules were detected, and sloughing of immature spermatogenic cells into the lumen was rare and minimal (Fig. 1D and E). Notably, in Treatment 6, no immature seminiferous tubules or sloughed spermatogenic cells were observed (Fig. 1F).

#### 3.2.2 Histology of cauda epididymis

**Fig. 2:**
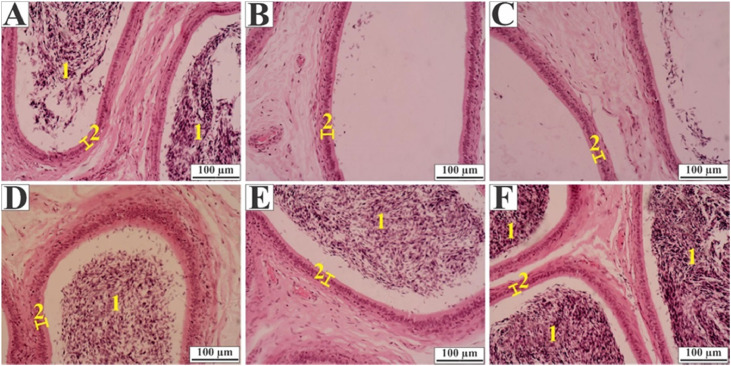
Effects of selenium sources and Lysophospholipid on histological section of epididymal tail tissue. A: Treatment 1. Control (basal diet + 0.5 mg of organic selenium per kg of DM); B: Treatment 2. Control diet + 0.05% Lysophospholipid; C: Treatment 3. Control diet + 0.1% Lysophospholipid; D: Treatment 4. Basal diet + 0.5 mg Nano selenium per kg of DM; E: Treatment 5. Basal diet + 0.05% Lysophospholipid + 0.5 mg of Nano selenium per kg of DM; F: Treatment 6. Basal diet + 0.1% Lysophospholipid to + 0.5 mg of Nano selenium per kg of DM. Hematoxylin-eosin staining. Magnification × 100. number 1- accumulation of sperm cells; Number 2- Epithelium covering the tubes of the tail part of the epididymis.

Histological analysis of the cauda epididymis showed that most tubules in Treatment 1 contained dense accumulations of sperm in the lumen (Fig. 2A). In contrast, Treatments 2 and 3 exhibited numerous epididymal tubules with little or no sperm content (Fig. 2B and C). Treatments 4 and 5 demonstrated predominant sperm accumulation in most tubules (Fig. 2D and E). Finally, Treatment 6 showed a high density of sperm cells within the epididymal tubules (Fig. 2F). No other remarkable differences were observed.

### 3.3 Histomorphometric results

Histomorphometric analysis revealed a significant increase in epithelial height in Treatment 6 compared to the other experimental treatments (P < 0.05). The minimum and maximum epithelial thickness values were observed in Treatment 2 (74.01 µm) and Treatment 6 (102.8 µm), respectively. No significant differences in epithelial height were found among the other treatments (P ≥ 0.05) (Table 3). No significant differences were observed in the epithelial height of the cauda epididymis among treatments (P ≥ 0.05). The minimum and maximum epithelial heights in the cauda epididymis were recorded in Treatment 1 (control diet) (19.3 µm) and Treatment 2 (21.1 µm), respectively (Table 3). There were no significant differences in the diameter of seminiferous tubules among treatments (P ≥ 0.05) (Table 3). However, the diameter of the seminiferous tubule lumen was significantly reduced in Treatments 3 and 6 compared to the other groups (P < 0.05) (Table 3), while no significant differences were observed among the remaining treatments (P ≥ 0.05). The number of Sertoli cells in Treatment 6 was significantly higher than in all other treatments except Treatment 4 (P < 0.05). The highest and lowest Sertoli cell counts per seminiferous tubule were recorded in Treatment 6 (30.0 cells) and Treatment 3 (15.7 cells), respectively (Table 3). Leydig cell counts were significantly increased in Treatment 6 compared to the other treatments (P < 0.05). In contrast, Treatment 2 showed a significant decrease in Leydig cell number compared to the other groups (P < 0.05). The highest and lowest Leydig cell counts within a 50 µm radius circle were observed in Treatment 6 (25.7 cells) and Treatment 2 (11.2 cells), respectively (Table 3). The tubular differentiation index (TDI) was significantly higher in Treatment 6 compared to all other treatments (P < 0.05), with the highest and lowest values observed in Treatment 6 (89.2%) and Treatment 4 (70.7%), respectively (Table 3). The repopulation index (RI), representing the ratio of active to inactive spermatogonia, was significantly higher in Treatment 6 (81.5%) compared to the other treatments (P < 0.05) (Table 3). The spermatogenesis index (SI) was significantly higher in Treatments 6, 5, and 4 compared to the other treatments (P < 0.05), with the highest value observed in Treatment 6 (89.2%) (Table 3). Finally, the number of degenerated tubules was significantly reduced in Treatments 5 (5.7 per section) and 6 (5.5 per section) compared to the other treatments (P < 0.05). In contrast, Treatment 2 showed a significantly higher number of degenerated tubules (18.2 per section) compared to all other groups (P < 0.05) (Table 3).

**Table 3:** Effects of selenium sources and Lysophospholipid on histomorphometrical parameters of testicular tissue.

	Treatments *						P-value			
	1	2	3	4	5	6	SEM	Lyso	Nano-Se	Lyso* Nano-Se
Diameter of seminiferous tubule (μ)**	247.36	267.48	246.2	252.12	240.77	293.85	6.85	0.42	0.51	0.08
Epithelium height (μ)	82.54^bc^	74.01^c^	76.64^bc^	86.74abc	92.02^ab^	102.82^a^	2.51	0.26	<0.01	0.04
Diameter of seminiferous tubules central lumen (μ)	123.46^a^	118.52^ab^	73.76^c^	94.42abc	85.7^bc^	78.88^c^	4.89	<0.01	<0.01	0.05
Number of Sertoli cells (per seminiferous tubule)	17.25^b^	19.0^bc^	15.75^c^	24.75^ab^	22.5^bc^	30.0^a^	1.15	0.33	<0.01	<0.01
Number of Leydig cells (in a circle with a radius of 50 μ)	17.5abc	11.25^c^	15.5^b^	19.25abc	17.25abc	25.75^a^	1.16	0.01	<0.01	0.13
Number of degenerated tubes (at each section)	15.75^ab^	18.25^a^	15.0^ab^	15.0^ab^	5.75^b^	5.5^b^	1.44	0.19	<0.01	0.11
Epididymal tail epithelium height (μ)**	21.12	19.27	19.41	20.81	20.36	20.79	0.32	0.33	0.27	0.53
TDI (%)^1^	78.5^bc^	81.0^b^	77.0^bc^	70.75^c^	79.75^bc^	89.25^a^	1.28	<0.01	0.43	<0.01
RI (%)^2^	73.0^b^	72.0^b^	61.5^c^	67.0^bc^	79.0^ab^	81.5^a^	1.56	0.02	<0.01	<0.01
SI (%)^3^	73.5^c^	74.5^bc^	73.75^bc^	83.75^a^	85.0^a^	89.25^a^	1.49	0.36	<0.01	0.35

⁎ Treatments: 1. Control (basal diet + 0.5 mg of organic selenium per kg of DM), 2. Control diet + 0.05% Lysophospholipid, 3. Control diet + 0.1% Lysophospholipid, 4. Basal diet + 0.5 mg Nano selenium per kg of DM, 5. Basal diet + 0.05% Lysophospholipid + 0.5 mg of Nano selenium per kg of DM, 6. Basal diet + 0.1% Lysophospholipid to + 0.5 mg of Nano selenium per kg of DM.

a,b,c Means within a row with different superscripts are significantly different (P<0.05). 1.Tubular differentiation index (TDI) 2. Repopulation Index of active spermatogonia (RI) 3. Spermiogenesis index (SI)

⁎⁎ No lowercase letters mean no significant difference.

### 3.4 Sperm quality

**Fig. 3:**
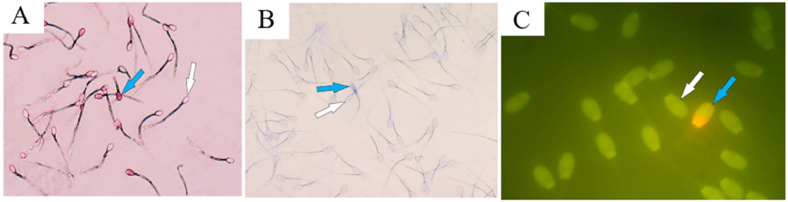
Effects of selenium sources and Lysophospholipid on Semen parameters. A: Eosin-nigrosin staining. White arrow indicates live sperm (sperm with white head) and blue arrow indicates dead sperm (sperm with red head); B: Aniline blue staining. White arrow indicates mature sperm (sperm with pale blue streak) and blue arrow indicates immature sperm (sperm with dark blue streak); C: Acridine orange staining. The white arrow indicates sperm with healthy DNA (sperm with green color) and the blue arrow indicates sperm with damaged DNA (sperm with yellow color).

There were no significant differences in mean semen volume among the experimental treatments. However, significant variations were observed in other parameters, as detailed below. The total sperm count in Treatment 6 (777.5 million/mL) showed a significant increase compared to all other treatments (P < 0.05). No significant differences were observed among the remaining treatments (Table 4). The percentage of live sperm in Treatment 6 (83.75%) was significantly higher than in the other treatments (P < 0.05). Conversely, Treatment 4 (61.25%) showed a significant decrease compared to the rest. No significant differences were detected among Treatments 1, 3, 4, and 5 (Table 4, Fig. 3A). The percentage of sperm with intact (normal) DNA was significantly higher in Treatment 5 (97.0%) compared to the other groups (P < 0.05). Treatment 4 also showed a significant difference compared to the rest. No significant differences were observed among Treatments 1, 2, 3, and 6 (Table 4, Fig. 3C). The percentage of sperm with histone-deficient immaturity was significantly lower in Treatment 6 (4.7%) than in the other treatments (P < 0.05). A significant difference was also observed in Treatment 4 compared to the others. No significant differences were found among Treatments 1, 2, 3, and 5 (Table 4, Fig. 3B). Sperm motility percentage was significantly increased in Treatments 4 and 6 (98.25%) compared to the other treatments (P < 0.05) (Table 4).

**Table 4:** Effects of selenium sources and Lysophospholipid on Semen parameters in Moghani rams.

	Treatments*						P-value			
	1	2	3	4	5	6	SEM	Lyso	Nano-Se	Lyso*Nano-Se
Total count of sperm (µl)	665.25^ab^	714.0^ab^	698.25^ab^	727.5^ab^	727.5^ab^	777.5^b^	11.86	0.21	<0.01	0.75
Sperm motility (% motile sperm)	95.25^b^	96.0^ab^	97.0^ab^	98.25^a^	98.25^a^	98.25^a^	0.28	0.05	<0.01	0.07
Sperm volume (ml)**	1.45	1.25	1.37	1.75	1.75	2.0	0.09	0.89	<0.01	0.61
Sperm viability (%)	75.25^b^	69.0^bc^	62.5^cd^	61.25^d^	61.25^d^	83.75^a^	1.81	0.02	<0.01	<0.01
Sperm with intact DNA (%)	94.0^ab^	92.25^ab^	93.0^ab^	88.0^b^	88.0^b^	93.75^ab^	0.81	0.1	0.9	0.01
Inexistency sperms histone immaturity(%)	6.25abc	9.5^bc^	8.25abc	10.0^a^	10.0^a^	4.75^c^	0.51	0.14	0.34	<0.01

⁎ Treatments: 1. Control (basal diet + 0.5 mg of organic selenium per kg of DM), 2. Control diet + 0.05% Lysophospholipid, 3. Control diet + 0.1% Lysophospholipid, 4. Basal diet + 0.5 mg Nano selenium per kg of DM, 5. Basal diet + 0.05% Lysophospholipid + 0.5 mg of Nano selenium per kg of DM, 6. Basal diet + 0.1% Lysophospholipid to + 0.5 mg of Nano selenium per kg of DM.

a,b,c,d Means within a row with different superscripts are significantly different (P<0.05).

⁎⁎ No lowercase letters mean no significant difference.

## 4 Discussion

The present study demonstrates that dietary supplementation with nano-Se and LPLs exerts dose-dependent and function-specific effects on reproductive performance in Moghani rams. Although no significant differences were observed in testicular weight or scrotal circumference among treatments (P ≥ 0.05), marked improvements were detected in spermatogenesis indices, histomorphometric parameters, and sperm quality traits. This apparent discrepancy highlights that macroscopic parameters are relatively insensitive indicators of early or moderate changes in testicular function, whereas cellular and histological indices—such as Sertoli and Leydig cell numbers, epithelial height, and spermatogenesis indices—are more sensitive markers of functional activity within the testes. Similar findings have been reported in ruminants, where nutritional or antioxidant interventions enhanced spermatogenesis without affecting testicular size (Hotzel et al., 1998; Abi Saab et al., 1997).

The most pronounced improvements in testicular structure and spermatogenic efficiency were observed in Treatment 6 (0.1% LPL + nano-Se), as evidenced by increased epithelial height, Sertoli and Leydig cell populations, and higher values of TDI, RI, and SI. These findings suggest that higher levels of LPL, when combined with nano-Se, enhance the testicular microenvironment and cellular proliferation, likely through improved nutrient absorption and enhanced antioxidant capacity. Selenium plays a critical role in male reproduction through its incorporation into selenoproteins such as glutathione peroxidase, which protect germ cells from oxidative damage and support spermatogenesis (Hosnedlova et al., 2018; Khalil et al., 2019). The nano-form of selenium has been shown to exhibit higher bioavailability and biological activity, leading to improved reproductive outcomes compared to conventional selenium sources (Arshad et al., 2021; Yuan et al., 2024).

In contrast, Treatment 5 (0.05% LPL + nano-Se) was more effective in improving functional sperm parameters, including total sperm count, viability, and DNA integrity. This suggests that lower LPL inclusion levels may preferentially enhance sperm maturation and functional competence, possibly by stabilizing sperm membranes and reducing oxidative damage during epididymal transit. Lysophospholipids (LPLs) are known to modulate membrane fluidity and permeability, which are essential for sperm motility and fertilization capacity (Joshi et al., 2006; Zhao et al., 2017). Recent studies have also demonstrated that LPL supplementation improves nutrient utilization and cellular metabolism, which may indirectly support sperm quality (Liu, Deng, Wang and Dong, 2023). Collectively, these findings indicate that different LPL inclusion levels exert complementary effects, with higher doses supporting spermatogenic structure and lower doses enhancing semen quality.

Interestingly, some treatment groups—particularly those receiving lower LPL levels without optimal interaction with nano-Se—showed reduced spermatogenesis indices and decreased seminiferous tubule lumen diameter. These responses may reflect dose-dependent or interaction effects, as bioactive compounds often exhibit non-linear relationships with physiological responses. Suboptimal supplementation may transiently affect germ cell proliferation or tubular morphology without necessarily impairing overall reproductive function. Moreover, a reduction in lumen diameter may represent adaptive changes in seminiferous tubules, such as increased density of germ cells or reduced degeneration, rather than a detrimental effect. Similar morphological variations have been observed in studies evaluating nutritional modulation of testicular function (Nikravesh and Jalali, 2004).

The improvement in sperm motility, viability, and DNA integrity observed in nano-Se–supplemented groups is consistent with the well-established role of selenium in mitigating oxidative stress. Excessive ROS production is known to impair sperm membrane integrity, reduce motility, and induce DNA fragmentation (Tvrdá et al., 2011; Agarwal et al., 2021). In the present study, although sperm motility was numerically higher than viability in some treatments, these differences were not statistically significant (P ≥ 0.05) and should therefore be interpreted with caution. Under physiological conditions, sperm motility and viability are closely associated, as only viable spermatozoa can exhibit sustained motility. Minor discrepancies between these parameters may arise due to methodological differences or transient alterations in membrane function (Dutta et al., 2019).

Despite the absence of significant changes in testicular size, the observed improvements in spermatogenesis can be attributed to enhanced cellular efficiency rather than tissue hypertrophy. Increased Sertoli cell numbers may improve support for germ cell development, while enhanced Leydig cell populations may promote androgen production, both of which are critical for spermatogenesis. These findings reinforce the concept that functional enhancement of testicular activity can occur independently of gross morphological changes, particularly under nutritional interventions targeting oxidative balance and cellular metabolism.

### Limitations and future perspectives

While the present study provides valuable insights, several limitations should be acknowledged. The relatively small sample size (n = 6 per treatment) may have limited the statistical power to detect subtle differences in some parameters. To mitigate this, multiple replicates, repeated semen sampling, and robust statistical analyses were employed. Additionally, the study duration (90 days) may not fully capture long-term reproductive responses. The use of a single breed and fixed supplementation levels also limits the generalizability of the findings. Nevertheless, standardized experimental conditions and validated analytical methods ensured the reliability of the results. Future studies with larger sample sizes, extended durations, and a broader range of supplementation levels are warranted to further elucidate the dose-response relationships and underlying mechanisms.

Overall, the present findings indicate that nano-Se and LPLs exert dose-dependent and complementary effects on ram reproductive performance. Higher LPL levels in combination with nano-Se primarily enhance testicular structure and spermatogenic efficiency, whereas lower LPL levels appear more effective in improving functional semen quality. These results provide a basis for optimizing nutritional strategies aimed at improving male fertility in sheep.

## 5 Conclusion

The present study demonstrates that dietary supplementation with nano-Se and LPLs can effectively improve reproductive performance in Moghani rams through dose-dependent and function-specific mechanisms. Although no significant changes were observed in testicular weight or scrotal circumference, substantial improvements were detected at the cellular and functional levels, indicating that testicular activity can be enhanced independently of gross morphological traits.

The combination of nano-Se with a higher level of LPL (0.1%) was more effective in improving testicular histomorphometry and spermatogenic efficiency, as reflected by increased Sertoli and Leydig cell populations and enhanced spermatogenesis indices. In contrast, the lower LPL level (0.05%) combined with nano-Se was more effective in improving functional semen quality, including sperm count, viability, and DNA integrity. These findings suggest that different supplementation strategies may be optimal depending on whether the goal is to enhance spermatogenic structure or sperm functional competence.

Overall, the results indicate that nano-Se and LPL exert complementary effects on male reproductive physiology, likely mediated through improved antioxidant status and modulation of cellular and membrane functions. However, given the relatively small sample size and fixed supplementation levels, further research with larger populations, extended experimental durations, and a wider range of doses is required to confirm these findings and refine practical recommendations for reproductive management in sheep.

## Data availability statement

The data that support the findings of this study are available from the corresponding author upon reasonable request.

## Funding statement

This research did not receive any specific grant from funding agencies in the public, commercial, or not-for-profit sectors.

## Ethics statement

All experimental procedures involving animals were conducted in accordance with the guidelines for the care and use of laboratory animals and were approved by the Ethics Committee of the University of Mohaghegh Ardabili (UMA), under approval number IR.UMA.REC.1404.056 (dated June 25, 2025). The approval was issued in the name of Leila Aghazadeh (PhD student) and Farzad Mirzaei Aghjeh-geshlagh (supervisor). All efforts were made to minimize animal suffering and to adhere to ethical standards in animal research.

## CRediT authorship contribution statement

**Leila Aghazadeh:** Conceptualization, Writing – original draft, Methodology. **Farzad Mirzaei Aghjeh-gheshlagh:** Data curation, Supervision. **Hossein Jahani-Azizabadi:** Formal analysis, Writing – review & editing. **Bahman Navid-Shad:** Investigation, Validation. **Ali Kalantari-Hesari:** Formal analysis. **Hamid Staji:** Writing – review & editing.

## Declaration of competing interest

The authors declare that they have no known competing financial interests or personal relationships that could have appeared to influence the work reported in this paper.
